# Successful Utilization of a Live Donor Kidney with Angiomyolipoma

**DOI:** 10.7759/cureus.6937

**Published:** 2020-02-10

**Authors:** George Rofaiel, Gilbert Pan, Jeffrey Campsen, Robin Kim, Blake Hamilton

**Affiliations:** 1 Surgery, University of Utah School of Medicine/Huntsman Cancer Institute, Salt Lake City, USA; 2 Urology, University of Utah School of Medicine, Salt Lake City, USA

**Keywords:** angiomyolipoma, kidney transplantation, live donor kidneys

## Abstract

The gap between the kidney transplant recipient list and the number of organs available for transplantation continues to grow. Kidneys from living donors are a major source of high-quality organs. However, they commonly have benign conditions such as cysts and benign tumors that present as operative challenges. This case presents a donor kidney that had a benign angiomyolipoma. The kidney was donated in a standard, minimally invasive fashion. The tumor was then removed on the back table and transplanted without an issue. Both donor and recipient enjoyed a speedy recovery with no significant complications.

## Introduction

There is a large discrepancy between the number of organ donors and the number of patients on the kidney transplant list [1]. As of December 10, 2019, more than 95,000 patients are on the waiting list for a kidney transplant in the United States [2]. The growth in kidney transplantation has been sluggish with only 21,000 cases performed in 2018 [3]. The number of living-donor procedures performed has also remained stagnant for several years with approximately 7,000 cases annually [2,4]. Patients on dialysis who are unable to receive transplants face high mortality and morbidity related to end-stage renal disease [5]. Kidneys donated by living donors are high-quality organs that afford longer graft survival for recipients [6]. The recovery of recipients also tends to be faster with lesser chance of delayed graft function [7].

Occasionally, donors present with benign conditions that can seem prohibitive to donations. One example is angiomyolipomas (AML), which are benign tumors that can grow to large sizes. AML is only second to renal cell carcinoma in causing spontaneous retroperitoneal hemorrhage [8,9]. Fortunately, a diagnosis can easily be made using contrast computed tomography (CT) scans that are normally utilized in evaluating living donors [10]. Case reports of transplanting kidneys with AML are sparse. Less common are the ones that report ex vivo back table resections.

## Case presentation

**Figure 1:**
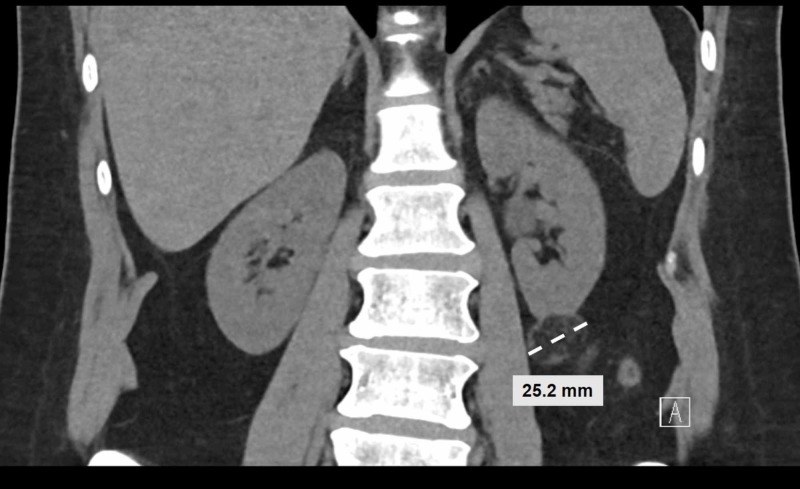
Angiomyolipoma identified from CT imaging

A healthy 54-year-old female presented to donate a kidney to a family member and was evaluated according to our standard protocol. Her CT scan showed a 2.52 cm left lower pole exophytic AML (Figure 1). Our team became concerned about leaving her with this tumor and putting her at risk for spontaneous rupture or growth of tumor in the solitary kidney. We were also concerned about further growth of the tumor in the recipient and risk of spontaneous rupture after transplantation. Given the associated risks, we decided to do an ex vivo excision of the tumor.

**Figure 2:**
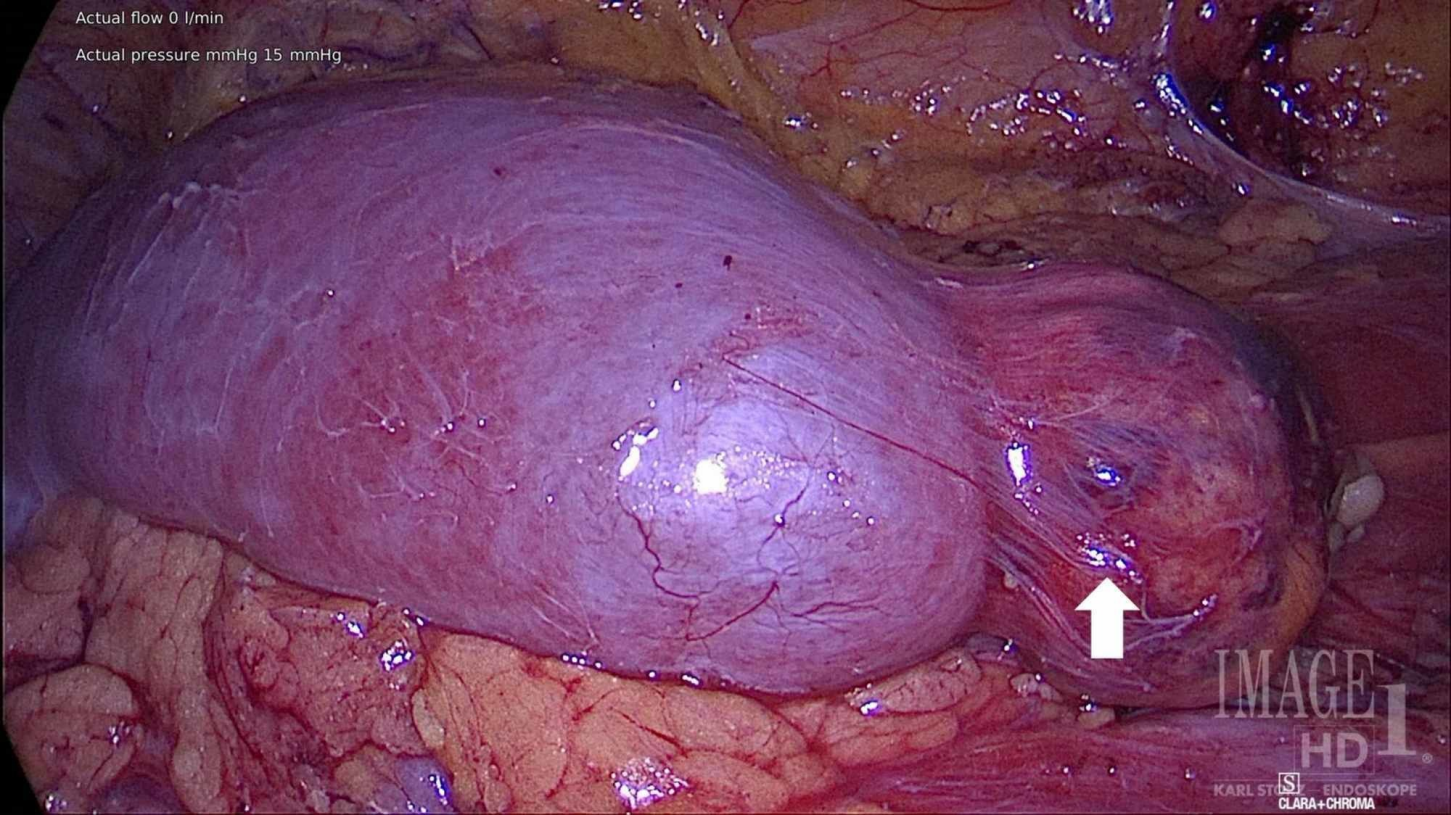
Intraoperative imaging of renal angiomyolipoma (white arrow) on lower pole of donor kidney

**Figure 3:**
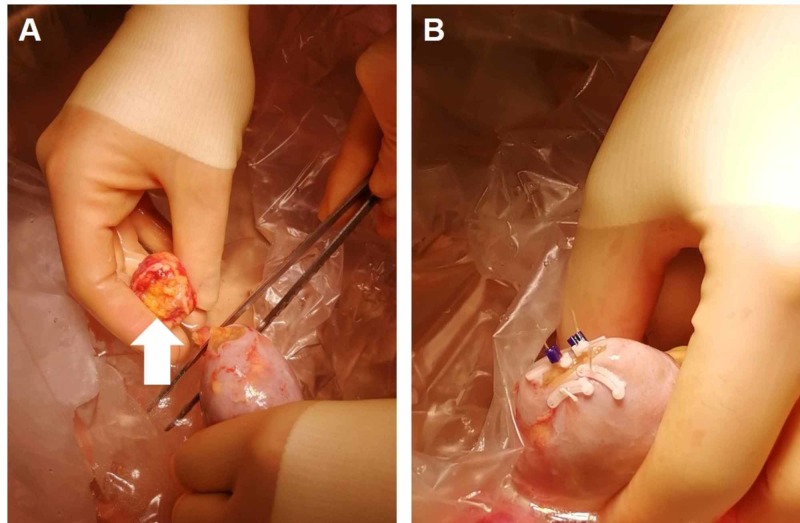
Ex vivo back table resection of angiomyolipoma from donor kidney (A) Excision of angiomyolipoma (white arrow) from lower pole of donor kidney followed by (B) closure of renal parenchyma

**Figure 4:**
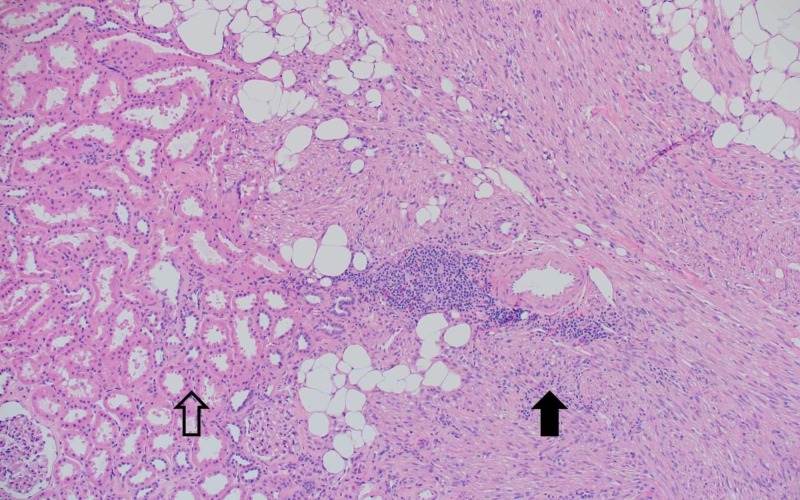
Native kidney (hollow arrow) transitioning to the three composite tissues of angiomyolipoma (solid arrow): dystropic vessels, adipose tissue, and smooth muscle

The donor kidney was removed in a standard hand-assisted laparoscopic donor nephrectomy procedure. During the operation, the tumor was easily seen and protected (Figure 2). The kidney was removed after dividing the vessels using a standard laparoscopic stapler. It was then flushed on the back table using histidine-tryptophan-ketoglutarate solution. The back table operation started with dissection of the vessels and ureter in the usual fashion. The urology team then stepped in to perform a partial nephrectomy. The tumor was cut sharply using a knife and resected. The parenchyma was closed using interrupted absorbable stitches (Figure 3A, 3B). The tumor specimen was later histologically confirmed to be a renal AML by surgical pathology (Figure 4).

The recipient operation proceeded with a standard open technique. A right lower quadrant incision was performed, and the iliac artery and vein were anastomosed to the corresponding iliac vessels. Upon reperfusion, the kidney showed minimal to no signs of bleeding from the excision site. Postoperatively, the recipient had immediate function of the graft. The recipient showed no signs of bleeding or urine leaks. Creatinine clearance increased appropriately to 91 ml/min by day 5 post-transplantation. At two months post-transplantation, the recipient continues to enjoy excellent renal function and reports no complications. 

## Discussion

Flum and colleagues reported on the overall management of patients with AML tumors [9]. The diagnosis seems to be made easily via CT scans because these tumors are generally fat rich, which gives them a characteristic appearance on a CT scan [10]. In less common scenarios, AML can be fat poor, and this represents a diagnostic challenge that can often be resolved using magnetic resonance imaging to visualize the mass [11]. It is rare for these tumors to require a biopsy or be diagnosed post excision [12]. Given their tendency to hemorrhage, the general consensus is that tumors should be excised electively when they are larger than 4 cm [13,14].

Niemi and Mandelbrot reported comprehensively on the utilization of medically complex patients for kidney donations and concluded that the use of complex patients as donors is worthwhile for recipients [7]. There is minimal reporting on the utilization of kidneys with AML tumors. Although, recently, Zahran and colleagues reported a small series of cases where donor kidneys with AML were utilized, they deemed the outcome to be good and safe for both the donor and the recipient [8].

Patients who present with small AML masses normally do not need intervention. However, should these patients choose to donate a kidney, leaving the benign AML mass puts the recipients at risk of bleeding or needing additional intervention. The natural history of AML after transplantation is unknown. The advantage of ex vivo excision is that all associated risks are managed and reduced in advance. This rationale is what led to our planned intervention. We had an excellent outcome where neither the donor nor recipient will need surveillance or follow-up in regard to AML.

## Conclusions

AML tumors are usually harmless, but can grow and hemorrhage spontaneously. The utilization of these kidneys after an ex vivo excision allows donors to help intended recipients receive very valuable organs. This also allows them not to worry about the benign tumor for their lifetime. On the recipient end, a large number of high-quality life years can be added. Removing the AML also allows recipients to not to worry about the risks of tumor-related complications. 
